# Lymph Node Involvement in Axillary Hidradenitis Suppurativa: A Clinical, Ultrasonographic and Bacteriological Study Conducted during Radical Surgery

**DOI:** 10.3390/jcm10071433

**Published:** 2021-04-01

**Authors:** Silvia Vaienti, Gianluca Nazzaro, Anna Grancini, Paolo Calzari, Giovanna Zaccaria, Stefano Veraldi, Luca Vaienti

**Affiliations:** 1 Department of Pathophysiology and Transplantation, Università degli Studi di Milano, Foundation IRCCS, Ca’ Granda Ospedale Maggiore Policlinico, Via Pace 9, 20122 Milan, Italy; silvia.vaienti@hotmail.it (S.V.); pcalzari@gmail.com (P.C.); stefano.veraldi@unimi.it (S.V.); 2 Dermatology Unit, Foundation IRCCS, Ca’ Granda Ospedale Maggiore Policlinico, Via Pace 9, 20122 Milan, Italy; 3 Microbiology Laboratory, Foundation IRCCS, Ca’ Granda Ospedale Maggiore Policlinico, Via San Barnaba 8, 20122 Milan, Italy; anna.grancini@policlinico.mi.it; 4 Division of Plastic Surgery, University of Modena and Reggio Emilia, Largo del Pozzo 71, 41124 Modena, Italy; zaccariagiovanna1982@gmail.com; 5 Department of Biomedical Sciences for Health, Università degli Studi di Milano, IRCCS Policlinico San Donato, Piazza Edmondo Malan 1, San Donato Milanese, 20097 Milan, Italy; luca.vaienti@unimi.it

**Keywords:** hidradenitis suppurativa, lymph node, ultrasound, microbiology, plastic surgery

## Abstract

Background: Hidradenitis suppurativa (HS) is an inflammatory and chronic-recurrent disease of the hair follicle. Its aetiopathogenesis is not completely known. Although bacterial colonization and superinfection are clinically relevant, lymph node involvement has rarely been studied. Objectives: In this pilot retrospective study, we evaluated the clinical and microbiological nodal involvement in patients with axillary hidradenitis suppurativa. Materials and methods: We retrospectively analyzed patients suffering from axillary hidradenitis suppurativa and referred to the Dermatology Unit of the Foundation Ca’ Granda Ospedale Maggiore Policlinico in Milan between October 2018 and November 2019. The sampling procedure took place during the surgical excision of lesions at the Operative Unit of Plastic and Reconstructive Surgery of Policlinico San Donato Hospital. Three types of sample were sent to microbiological analysis: exudate swab, axillary lymph node, fistula. Result: In total, we recruited 10 patients. Two of them underwent bilateral axillary surgery. Nine lymph nodes were analyzed. In one patient, bacterial culture in lymph nodes, skin lesions and fistulae matched. Conclusions: Unequivocable conclusions cannot be drawn due to the low number of patients. Further studies are necessary to confirm the preliminary results of our retrospective pilot study.

## 1. Introduction

Hidradenitis suppurativa (HS) is a chronic, inflammatory, recurrent and debilitating disease of the hair follicle that usually presents with painful abscesses and sinus tracts involving the apocrine glands bearing skin, such as the axillary, inguinal and anogenital regions [1,2].

The disease is commonly classified according to the Hurley score [3].

The diagnosis is clinical and can be confirmed by ultrasound examination [4,5,6].

Currently, the aetiology of HS is not completely clarified. Bacterial colonization seems to play an important role. In most cases, bacteria identified only partially correspond to the normal skin microflora [7,8].

While cutaneous bacterial colonization has been studied extensively, little is reported about bacterial invasion into deep tissues and the involvement of draining lymph nodes [9,10].

This retrospective pilot study aims to evaluate the involvement of draining lymph nodes in patients with axillary hidradenitis suppurativa. These data were compared to those found in more superficial areas.

## 2. Materials and Methods

### 2.1. Patients

After institutional review board approval and respecting the 1975 declaration of Helsinki, we retrospectively analyzed patients suffering from axillary HS and referred to Dermatology Unit of the Foundation Ca’ Granda Ospedale Maggiore Policlinico in Milan between October 2018 and November 2019. Data were managed anonymously, and specific informed consent was obtained from every patient.

Inclusion criteria were: Moderate–severe (Hurley stage II–III) axillary HS, radical surgical excision of the entire hair-bearing skin of the axilla.

Exclusion criteria were: Involvement of areas other than axilla, antibiotic treatment prescribed in the previous month. Patients with inguinal-genital and gluteal HS were excluded because, in these areas, lymph node reactivity and risk of bacterial contamination are generally higher.

Two patients out of 10 underwent bilateral radical excision.

For each subject, before the surgical procedure, lymphadenopathy was evaluated on physical and on ultrasonographic examination.

### 2.2. Samples

The radical surgical excision was performed at the Operative Unit of Plastic and Reconstructive Surgery of Policlinico San Donato Hospital.

An exudate swab was taken prior to the sterile prepping. After completion of the surgical excision, a deep sinus tract and a sample of draining lymph nodes were harvested from the specimen. All the three samples were sent into a sterile container for microbiological analysis.

### 2.3. Bacteriological Examinations

The microbiological samples (lymph node, exudate swab, fistula) were incubated following standard procedures in aerobic and anaerobic conditions (Table 1).

Identification of the bacterial species was performed using Matrix Assisted Laser Desorption Ionization Time-of-Flight (MALDI-TOF^®^).

## 3. Results

### 3.1. Clinical and Ultrasound Data

In total, we recruited 10 patients.

Lymphadenopathy was not detected in any patient at the clinical examination.

Hyperplastic lymph nodes were detected in two patients on ultrasound.

These data are shown in Table 2.

### 3.2. Microbiological Data

Two out of patients underwent bilateral axillary surgery.

In total, nine lymph nodes were analyzed. The diameter range of the nodes was between 0.4 and 1 cm.

In three cases, lymph nodes sampling was not possible because of their small size. Bacteriological examinations of lymph nodes, exudate swabs and fistula tracts were available in 7, 8 and 9 patients, respectively.

All samples presented with colonizing bacteria, except for two lymph nodes (Table 3).

In one patient, bacteriological culture revealed the same bacterium (*Enterococcus faecalis*) in lymph nodes, skin lesions and fistulae.

Microbiological data are compared in Table 3. In this table, all the bacterial species cultured are shown.

## 4. Discussion

Only a few studies in the English literature focused on lymph node involvement in HS [9,10], and most were ultrasonographic. Wortsman [9] performed ultrasound examination in patients in Hurley stage II and III in the axillary and inguinal areas. In this study, a transverse lymph node diameter ≥1 cm was used as a parameter to define lymphadenopathy. No significant difference was detected between the measurement of pathological cases in stage II and controls. However, a slight discrepancy was observed between stage III cases and controls. It was hypothesized that the lymph node involvement could reflect the presence of a secondary infection, without having a primitive etiological role.

In a previous article [10], we demonstrated an ultrasonographic involvement of axillary lymph nodes in 23,3% of Hurley II and in 63% of Hurley III patients.

Nesmith, observing the high rate of surgical re-operation due to infections and wound dehiscence, hypothesized that this could be related to the permanence of infected tissue, including lymph nodes, not included in the area of excision [11]. The authors conducted the research on 11 patients (for a total of 15 procedures) who underwent radical excision of axillary lesions, associated with lymphadenectomy of the axillary lymph node level. In 93% of cases, lymphadenopathy was detected and lymphadenectomy was performed to prevent relapse: Nesmith concluded that en bloc removal associated with lymphadenectomy was both a curative solution with low comorbidity and could be used as a guide to set up specific post-operative antibiotic therapy.

In our study, the radical excision allowed deep samples to be obtained, limiting the contamination of tissues. Methods such as needle aspiration or skin swab, commonly used in microbiologic studies, present a higher possibility of contamination.

At intraoperative measurement, an increase in lymph node size was never detected (the maximum diameter was 1 cm) and, in three cases, sampling was not possible due to the low volume of axillary lymph nodes. These data may indicate the uninvolvement of lymph nodes in HS, but culture tests appear mostly positive and heterogeneous. The detected bacteria are both typical of the normal cutaneous microflora (for example, *Staphylococcus epidermidis* and *Corynebacterium* spp.) and atypical (for example, some species typical of the intestinal environment such as *Proteus mirabilis*, *Escherichia coli*, *Enterobacter aerogens*, *Enterococcus faecalis*).

## 5. Conclusions

Therefore, drawing unequivocable conclusions is not possible because of the low number of patients recruited. This aspect represents the main limitation of our pilot retrospective study. Lymph nodes are clinically and ultrasonographically uninvolved, although abscesses and inflammatory lesions in axillary skin areas, but most of the lymph node culture tests are positive.

Further studies with larger populations are necessary in order to confirm our preliminary results.

The authors certify that they obtained consent forms from patients. In the form, each patient gave her/his consent for her/his clinical information to be reported in the study.

## Figures and Tables

**Table 1:** Culture Media and Incubation Times.

Culture Medium	Incubation Modalities
Chocolate agar	24–48 h
Sheep blood agar 5%	24–48 h
Mannitol salt agar	35–37 °C for 24 h
MacConkey agar	35–37 °C for 24 h
Sabouraud Dextrose Agar	32 °C for 5 days, reading after 48 h
Sheep blood Schaedler agar 5%	Anaerobic atmosphere 35–37 °C for 48 h
Sheep blood Schaedler agar 5% with Neomycin–Vancomycin	Anaerobic atmosphere 35–37 °C for 48 h
Columbia agar with sheep blood agar 5% with nalidixic acid and colistin.	Anaerobic atmosphere 35–37 °C for 48 h

**Table 2:** Clinical and ultrasound patients’ characteristics.

Total Number of Patients	10
Clinical stadion (Hurley)	
• I	0
• II	5
• III	5
Ultrasound stadiation (SOS-HS)	
• I	0
• II	3
• III	7
Lymphadenopathy (clinical examination)	
• Present	0
• Absent	10
Lymphadenopathy (ultrasound examination)	
• Present	2
• Absent	8

Moderate–severe (Hurley stage II–III).

**Table 3:** Summary and comparative table of the results obtained in the different cultural examinations.

		Bacteriological Culture Test		
		Lymph Node	Exudate Swab	Fistula
Patient 1		Negative	*Parvimonas micra*	*Pseudomonas aeruginosa*
Patient 2	Axilla A	Negative	*Proteus mirabilis*	*Proteus mirabilis*
	Axilla B	*Staphylococcus epidermidis* Anaerobic bacteria Gram neg.	Anaerobic bacteria Gram neg.	*Staphylococcus epidermidis*
Patient 3		*Enterobacter aerogens*	*Staphylococcus* spp. Mixed anaerobic bacterial flora	*Staphylococcus* spp. *Corynebacterium* spp. *Streptococcus* spp. Mixed anaerobic flora
Patient 4		*Staphylococcus epidermidis*	Abundant mixed flora	*Enterococcus faecalis*
Patient 5		*Enterococcus faecalis*	*Enterococcus faecalis* *Escherichia coli*	*Enterococcus faecalis*
Patient 6		Sampling not possible	*Staphylococcus aureus*	*Staphylococcus aureus*
Patient 7	Axilla A	*Proteus mirabilis*	Data not available	Data not available
	Axilla B	*Proteus mirabilis*		*Proteus mirabilis*
Patient 8		Mixed anaerobic bacterial flora	*Peptoniphilus asaccharolyticus* *Finegoldia magna*	Mixed anaerobic bacterial flora
Patient 9		Sampling not possible	*Stapylococcus homins* spp. *homins*	*Stapylococcus homins* spp. *homins*
Patient 10		Sampling not possible	Data not available	*Stapylococcus aureus*

## Data Availability

Data is contained within the article.
